# Evidence and Evidence Gaps in Adolescent Health

**DOI:** 10.1016/j.jadohealth.2016.08.001

**Published:** 2016-10

**Authors:** George Patton, Marleen Temmerman

**Affiliations:** Centre for Adolescent Health, Murdoch Childrens Research Institute, Melbourne, Australia; Department of Paediatrics, University of Melbourne, Melbourne, Australia; Centre of Excellence in Women and Child Health, Aga Khan University, Nairobi, Kenya; International Centre for Reproductive Health, Ghent University, Ghent, Belgium

The momentum to bring adolescents and young adults to center stage in global health and international development is palpable. Adolescents are increasingly seen as a crucial group for the success of the newly adopted Agenda for Sustainable Development [1]. Sitting within the Agenda for Sustainable Development framework, the 2030 Global Strategy for Women's, Children's and Adolescents' Health has extended the Every Woman, Every Child agenda to adolescence [2]. The strategy articulates the need for adolescent responsive health systems as well as social determinants, a focus that extends to legal and policy environments [3]. Countries seeking to adopt this more holistic approach to adolescent health and human rights must extend their public health efforts beyond the traditional yet still essential focus on HIV and sexual and reproductive health to address other infectious diseases, injuries, undernutrition, violence, self-harm, mental health, and the prevention of risks for noncommunicable diseases.

Good adolescent health generates a “triple dividend” from optimal growth and fulfilled youth potential, healthier trajectories across the life course and the healthiest possible start to life for the next generation [4]. In addition, with the largest generation of 10- to 24-year-olds in human history, the benefits will never be greater. Yet there are limits to our current capacity to respond [4]. Service delivery systems remain poorly developed. Equally, systems for collecting data on health need are weak and poorly coordinated so that few countries have a capacity to set balanced priorities or monitor progress. In addition, the coordination of health actions both within and outside the health service sector is weak. Arguably, the greatest limitations lie in the evidence for action. This supplement to the *Journal of Adolescent Health* addresses that challenge.

The first article in this supplement, “Adolescent Health and Well-Being: Background and Methodology for Review of Potential Interventions,” frames adolescence and adolescent health in the life course and intergenerational cycle [5]. Many mental and substance use disorders of adulthood emerge during these years [6]. So too, risk processes for the noncommunicable diseases of later life, including cardiovascular disease, cancer, and musculoskeletal and metabolic problems, commonly arise during adolescence [7]. Many risks for maternal and child health also begin during the adolescent and young adult years prior to first pregnancy. Because they affect fetal development before the initiation of antenatal care, the argument for preventive action during the adolescent and young adult years is compelling [8], [9].

The second article, “Improving Adolescent Sexual and Reproductive Health: A Systematic Review of Potential Interventions,” affirms the value of comprehensive sexuality education and provision of contraceptives in a setting of youth-friendly care [10]. Because we have had more evaluation of actions for sexual and reproductive health than other areas, the knowledge gaps are doubly striking. They include a lack of studies around female genital mutilation, child marriage, and sexual and intimate partner violence. Promising approaches such as the use of social and digital media to promote greater sexual health knowledge and contraceptive use have had little evaluation, perhaps in part because the technology has changed very quickly.

The third article in this supplement, “Interventions to Improve Adolescent Nutrition: A Systematic Review and Meta-Analysis,” reminds us of adolescence as a time of growth in all body systems, growth that is too often compromised by undernutrition, particularly for girls who become pregnant [11]. The significance of adolescent nutrition is even greater, given the potential for at least partial mitigation of the effects of early life stunting. Accelerating rates of obesity have further complicated nutritional risks arising in adolescence so that in many places, obesity coexists with food insecurity and undernutrition [12]. Most studies of adolescent nutrition have focused on pregnant girls. Even so, the evidence for effects on infant outcomes is still poor. There is greater clarity around responses to micronutrient deficiencies and iron deficiency anemia in particular. Yet despite findings that school-based delivery benefits iron stores and hemoglobin levels, adolescent iron deficiency anemia stubbornly persists in many low- and middle-income countries [13].

The supplement then turns to the subject of immunizations in “Systematic Review and Meta-Analysis of Interventions to Improve Access and Coverage of Adolescent Immunizations” [14]. The absence of evaluation studies is nowhere more striking than for vaccination. It is one of the most effective tools in public health, but the predominant focus has been on younger children. The opportunities for vaccination during adolescence are great both in completion of childhood schedules and for a range of primary immunizations. School-based vaccination within the context of national financing frameworks appears likely to be effective, but the need for further quality evaluation is clear.

Adolescence is the time of first episodes of most mental and substance use disorders of adulthood, the topic of the fifth and sixth articles in this supplement, “Interventions for Adolescent Mental Health: An Overview of Systematic Reviews” and “Interventions for Adolescent Substance Abuse: An Overview of Systematic Reviews” [15], [16]. Most intervention studies derive from high-income countries even though adolescents in low- and middle-income countries are equally affected by mental disorders. Studies of school-based interventions show promise, but there is a need to extend action beyond a dominant focus on cognitive behavioral therapy models. Substance use disorders are increasing in many parts of the world with links to violence, unintentional injury, HIV/STIs, and unwanted pregnancy. The predominant policy focus has been on legislation to restrict access and taxation to increase prices [4]. Yet as the article on substance use interventions suggests much more can be done, with school-based, family-focused, and digital platforms showing particular promise [16]. Coordinated multicomponent interventions seem likely to be the most effective strategy, but there are still few, quality evaluations.

Injuries and violence affect adolescents more than other age groups [17]. Adolescents are among the most vulnerable of road users and benefit disproportionately from injury control measures such as enforcing speed limits, introducing legislation around drink driving and helmets, improvements in road and motor vehicle design as well as effective trauma care. These strategies have been evaluated most extensively in high-income countries but are also likely to be effective elsewhere. The seventh article in this supplement, “Interventions to Prevent Unintentional Injuries Among Adolescents: A Systematic Review and Meta-Analysis,” reminds us that adolescent-targeted efforts are also of value with the evidence clearest around graduated licensing [18].

The method adopted in each of the accompanying reviews was an extended meta-review, with updates of older reviews and undertaking of some primary reviews in areas where none existed. The authors were therefore able to cover a huge area and scope the major evidence gaps. Some conclusions make sobering reading. The poor quality of evaluations and insufficient use of randomized controlled designs is clear. In areas such as mental disorders and sexual and intimate partner violence, we do not yet have effective and scalable strategies. There are a few interventions where costings are availability across diverse settings so that most available cost-effectiveness analyses are limited and unconvincing. Our available evidence base derives largely from high-income countries with few reports of feasibility in low- and middle-income countries. The success of interventions in girls as opposed to boys is often unclear, as is scalability across socioeconomic, socially marginalized, and ethnic groups. The final article in this supplement, “Adolescent Health Interventions: Conclusions, Evidence Gaps and Research Priorities,” addresses these limitations and recommends steps to mitigate these in the future [19].

There are also findings of great promise. Schools are an increasingly important platform for action. The most effective school actions are likely to be multicomponent and coordinated across different aspects of school life and activity [20]. With a growing retention of adolescents in secondary education in so many countries, the task of developing and scaling-up health actions within schools should become a priority. In many contexts, community-based intervention is an effective alternative and likely to remain important in places where rates of secondary school attendance are low.

The value of youth-friendly care is also clearer, particularly around sexual and reproductive health services [21]. In addition, there are new platforms with great potential, none more so than social and digital media. With the rollout of broadband in many countries, there is an unprecedented opportunity for innovation and evaluation to extend our knowledge of digital media interventions that currently derives from a few high-income countries.

Yet much more is needed for an adequate evidence base for global adolescent health. In low- and middle-income countries, a lack of evaluation studies and technical capacity has restricted progress. The absence of an international agency or network with responsibility for collecting and aggregating the evidence is a further factor. The Global Strategy for Women's, Children's and Adolescents' Health offers an unparalleled opportunity to extend the knowledge for action through evaluation, monitoring, and implementation research. Further demonstrations of effective action will be essential for securing a sustained investment from government and nongovernment sources into adolescent health in the future.
